# Sarcoidosis and its oral manifestations: A case report study

**DOI:** 10.1002/ccr3.6923

**Published:** 2023-02-10

**Authors:** Mehdi Shahabinejad, Zahra Delavarian, Toktam Zamani, Fatemeh Fallah Toosi

**Affiliations:** ^1^ Oral and Maxillofacial Diseases Research Center Mashhad University of Medical Sciences Mashhad Iran; ^2^ Oral and Maxillofacial Pathology Department Mashhad University of Medical Sciences Mashhad Iran; ^3^ Oral and Maxillofacial Medicine, Oral and Maxillofacial Diseases Research Center Mashhad University of Medical Sciences Mashhad Iran; ^4^ Oral and Maxillofacial Medicine Mashhad University of Medical Sciences Mashhad Iran; ^5^ Mashhad University of Medical Sciences Mashhad Iran

**Keywords:** dentistry, granulomatous diseases, medicine, pharmacology, sarcoidosis

## Abstract

A patient was referred to the oral medicine department with redness and swelling of the lips and cheek, and an intra‐oral lesion. Biopsy and laboratory investigations suggested a diagnosis of sarcoidosis. In this study we discuss oral findings associated with sarcoidosis.

## Abstract 2

As oral involvement may be the earliest manifestation of sarcoidosis in approximately two‐thirds of sarcoidosis patients, it is important for dentists to be aware of, and pay attention to common oral signs and symptoms. Moreover, diagnosis in this early stage may be associated with better prognosis.

## 1 INTRODUCTION

Sarcoidosis is a multisystem granulomatous disorder of unknown etiology in which T‐lymphocytes, mononuclear phagocytes, and granulomas destroy affected tissues.^1^ Boeck coined the term sarcoidosis (Greek meaning “flesh‐like condition”).^2^

The diagnosis of sarcoidosis is made through demonstration of non‐caseating granulomas in the biopsy sample in the presence of supporting clinical factors. Elevated serum angiotensin‐converting enzyme (ACE) levels could additionally support the diagnosis.^1^, ^2^, ^3^ Sarcoidosis displays increased prevalence in women.^2^ The disease may present acutely or follow a chronic course with periods of remission.

In approximately two‐thirds of patients, oral signs are the first manifestation of the disease. And the most commonly involved intra‐oral soft tissue sites are the buccal mucosa, gingiva, lips, tongue, and palate.^2^ Management options may include observation, systemic corticosteroids, and steroid‐sparing agents.^1^

In this study, we emphasize the oral features of sarcoidosis, a multi‐factorial disease potentially involving vital organs. As oral involvement may be the earliest manifestation of disease, it is important for dentists to be aware of, and pay attention to common oral signs and symptoms. Moreover, diagnosis in this early stage may be associated with better prognosis.

## 2 CASE PRESENTATION

**FIGURE 1:**
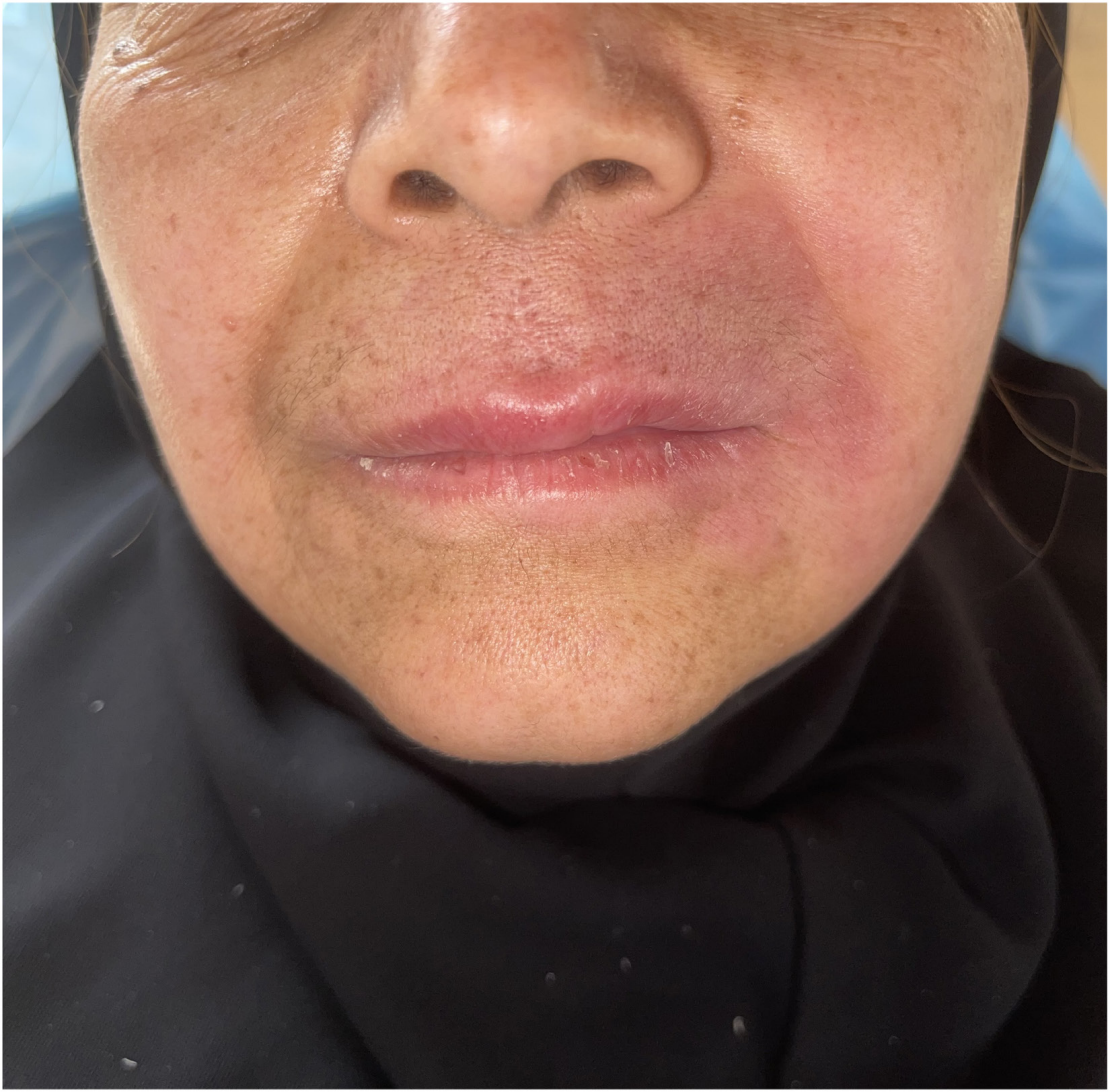
The extra‐oral view of the patient. Note the diffuse swelling of the lips and redness of the skin around the lips.

A 47‐year‐old woman was referred to the Oral and Maxillofacial department of the Mashhad University of Medical Sciences, with redness on the skin of her left cheek, and non‐tender diffuse swelling of the lips (especially the upper lip) appearing 5 months prior to referral (Figure 1). She declared that no improvement was obtained with prescription and use of Tetracycline ointment and Amoxicillin. Her medical records revealed a history of hypertension and hypothyroidism and usage of Captopril 250 mg (twice a day) and Levothyroxine 50 mcg (daily).

Extra‐oral examination demonstrated edematous inflammation of the upper lip with redness, particularly in the upper left area, mild swelling of the lower lip, and swelling and erythema on the skin of the left cheek. Asymmetry is clearly observed with lymphadenopathy in the left submandibular area with firm consistency but without tenderness on palpation.

An intra‐oral examination revealed a multi‐lobular exophytic lesion in the left buccal mucosa with a smooth surface, rubbery consistency, and normal color.

An initial diagnosis of OFG was made based on intra‐ and extra‐oral manifestations. Definitive diagnosis required rule‐out of other local and systemic factors, the first step to which is often a biopsy.

**FIGURE 2:**
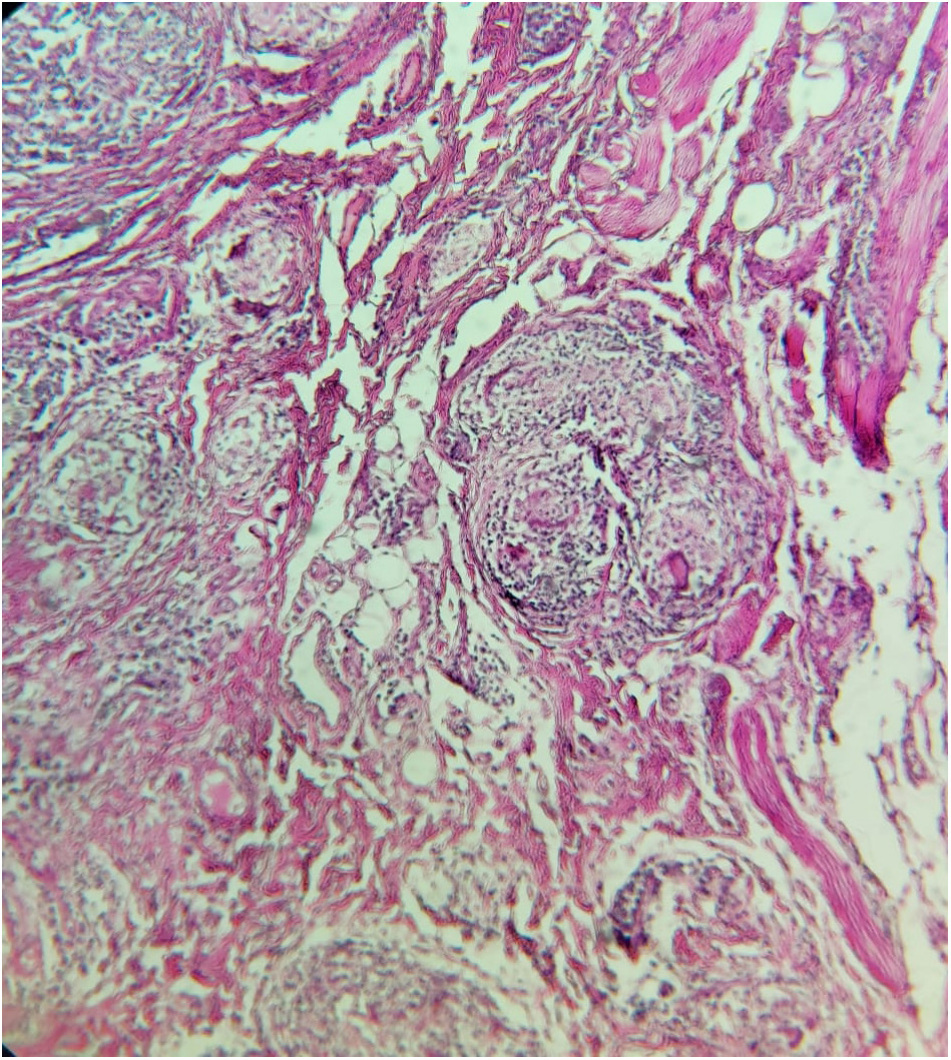
Microscopic view of giant cell bodies and Asteroid bodies and bundles of histiocytes.

Macroscopic features noted on the biopsy report included soft and elastic consistency as well as grayish color of the mucous membrane. Microscopic examination demonstrated granulomatous inflammation with bundles of histocytes surrounded by a lymphocytic rim, with observation of foreign body giant cells, and asteroid bodies with stellate inclusions (Figure 2). A diagnosis of Sarcoidosis was subsequently established.

Further laboratory investigation revealed a moderately high ACE level (78 IU/L) and a slightly higher than normal ESR level (25 mm/h), which supported a diagnosis of sarcoidosis. A chest x‐ray was unremarkable.

The patient was referred to a rheumatologist and subsequently prescribed 15 mgs prednisolone daily for 2 weeks, with further instruction for tapering over an additional 6 weeks in case of satisfactory response. Follow‐up examination in the third week demonstrated relative improvement. Unfortunately, cessation of treatment at 8 weeks was followed by the return of redness and swelling, and despite instructions for a repeat visit by a rheumatologist, the patient failed to present until after an additional 2 month. The repeat visit by the rheumatologist was further followed by consultation with a pulmonologist in consideration of the patient's complaints of single coughs beginning 1 month after initial presentation. Pulmonary investigation, including a computed tomography (CT) scan of the lungs, revealed no evidence of active pulmonary disease, and the patient received only symptomatic treatment. For unknown reasons however, the patient was not restarted on corticosteroids until a total of 6 months after cessation of the initial prednisolone course; 7.5 mgs prednisolone and 200 mgs hydroxychloroquine daily were prescribed upon the most recent rheumatology visit. Patient reported satisfactory results at follow‐up via phone call 3 weeks after start of the new regimen.

We strongly urge dentists to be aware of these oral manifestations of Sarcoidosis, as it may facilitate earlier diagnosis and more favorable outcomes.

## 3 DISCUSSION

Sarcoidosis is described as a disease in which the clinical signs and symptoms are typically not severe enough to cause alarm. Although the prognosis of sarcoidosis is generally favorable with a spontaneous regression rate of about 60%, it may nevertheless involve the heart, kidneys, central nervous system and lungs, with an overall case fatality rate of 4%–10%.^4^

Although soft tissue lesions of the oral cavity are not common occurrences in sarcoidosis,^5^ they may nevertheless be the patient's presenting complaint. In our case, the patient presented with a lesion on the buccal mucosa accompanied by edematous inflammation of the nearby skin.

Swelling of the cheek, buccal mucosa and lips, as in our case, suggest Orofacial Granulomatosis, the other signs of which may include Cobble‐stone swellings of oral mucosa or other evidence of focal submucosal enlargement, a grooved tongue, and gingival inflammation.^6^ A diagnosis of OFG requires systemic diseases such as Tuberculosis, Sarcoidosis, Crohn's disease, Melkersson‐Rosenthal syndrome, and Granulomatosis with polyangiitis to first be ruled out, however, and while signs more suggestive of an alternative diagnosis (e.g., occurrence of multiple nodules, xerostomia, and involvement of the salivary glands, common findings in the case of sarcoidosis) may provide diagnostic guidance, definitive diagnosis commonly requires microscopic examination of affected tissue.^7^, ^8^, ^9^, ^10^

The microscopic view of OFG typically demonstrates aggregates of non‐caseating granulomatous inflammations,^11^ a feature also characteristic of sarcoidosis; Asteroid and Schaumann bodies, however, are uncommon findings in OFG and, as in our case, their presence strongly suggests sarcoidosis.^12^

Measurement of serum angiotensin‐converting enzyme (ACE) levels can be helpful in both diagnosis and the monitoring of response to treatment, with raised levels in about 60–80% of patients with sarcoidosis.^12^, ^13^ Although, due to its poor specificity, an increase in ACE levels does not necessarily indicate the diagnosis of sarcoidosis.^14^ In our case, ACE levels were higher than the normal range (78 IU/L) which provided additional support for a diagnosis of sarcoidosis.

Further support may be provided by a rise in ESR levels.^15^ In our case, an elevation in ESR levels to 25 mm/h (with a laboratory reported upper limit of normal of 22) was not significant enough to warrant diagnostic consideration.

Treatment options for sarcoidosis may include expectant management, systemic corticosteroids, and/or surgical excision.^16^ Corticosteroids are generally considered beneficial in the acute phase of sarcoidosis, with oral glucocorticoids commonly being the first line of pharmacologic treatment.^17^ Accordingly, in our case, initial treatment consisted of the prescription of 15 mgs prednisolone daily.

An absence of treatment response is rare in sarcoidosis, and calls for verification of the absence of a diagnosis error.^18^ Relative treatment failure in our case appears to largely be a result of inadequate follow‐up and the patient's low adherence to treatment recommendations.

Nevertheless, studies have shown that oral sarcoidosis may be accompanied by more severe systematic involvement,^19^ and close follow‐up is recommended. We hope to bring awareness, to other dentists and fellow researchers, toward the oral signs and symptoms of sarcoidosis, features that are commonly assumed to be unimportant or irrelevant to the greater course of the disease.

## AUTHOR CONTRIBUTIONS

**Mehdi Shahabinejad:** Conceptualization; investigation; supervision; writing – review and editing. **Zahra Delavarian:** Investigation; project administration; supervision; writing – original draft; writing – review and editing. **Toktam Zamani:** Conceptualization; investigation; writing – original draft; writing – review and editing. **Fatemeh Fallah Toosi:** Investigation; writing – original draft; writing – review and editing.

## FUNDING INFORMATION

None.

## CONFLICT OF INTEREST STATEMENT

The authors declare no conflict of interest.

## CONSENT

I confirm that written patient consent has been signed and collected in accordance with the journal's patient consent policy.

## Data Availability

None.
